# Quantitative measurement of ^219^Rn radioactivity in exhaled breath from patients with bone metastasis of castration-resistant prostate cancer treated with ^223^RaCl_2_

**DOI:** 10.1186/s40658-019-0249-8

**Published:** 2019-07-26

**Authors:** Kazuhiro Ooe, Tadashi Watabe, Takashi Kamiya, Takashi Yoshimura, Makoto Hosono, Atsushi Shinohara, Jun Hatazawa

**Affiliations:** 1 Department of Nuclear Medicine and Tracer Kinetics, Osaka University Graduate School of Medicine, 2-2 Yamadaoka, Suita, Osaka, 565-0871 Japan; 2 Division of Science, Institute for Radiation Sciences, Osaka University, 1-1 Machikaneyama, Toyonaka, Osaka, 560-0043 Japan; 3 Division of Radiology, Department of Medical Technology, Osaka University Hospital, 2-15 Yamadaoka, Suita, Osaka, 565-0871 Japan; 4 Radioisotope Research Center, Institute for Radiation Sciences, Osaka University, 2-4 Yamadaoka, Suita, Osaka, 565-0871 Japan; 5 Institute of Advanced Clinical Medicine, Department of Radiology, Kindai University Faculty of Medicine, 377-2 Ohno-Higashi, Osaka-Sayama, Osaka, 589-8511 Japan; 6 Department of Chemistry, Graduate School of Science, Osaka University, 1-1 Machikaneyama, Toyonaka, Osaka, 560-0043 Japan; 7 Division of Education, Institute for Radiation Sciences, Osaka University, 1-1 Machikaneyama, Toyonaka, Osaka, 560-0043 Japan

**Keywords:** Radium-223, Radon-219, Breath of patients, Effective dose to caregivers

## Abstract

**Background:**

The α-emitting radionuclide radium-223 (^223^Ra) is widely used for the treatment of bone metastasis in patients with castration-resistant prostate cancer. However, ^223^Ra decays into radon-219 (^219^Rn) which is a noble-gas isotope, and ^219^Rn may escape from patients treated with ^223^Ra via their respiration. In this study, we quantified the amount of ^219^Rn contained in the breath of patients treated with ^223^Ra to estimate its effect on the internal exposure dose of caregivers.

**Methods:**

A total of 12 breath samples were collected using a breath collection bag from a total of six patients treated with ^223^RaCl_2_. Approximately 300 mL of exhaled breath was collected in a breath bag at 1 min and at 5 min after the start of ^223^RaCl_2_ administration. The contents of each bag were measured using an HPGe detector, and the amount of ^219^Rn was quantified based on the detection of the γ peak of ^211^Bi, which is a descendant nuclide of ^219^Rn, persisting in the breath bag. The effective dose to caregivers arising from the inhalation of ^219^Rn was estimated by referring to the scenario for the calculation of release criteria established for ^131^I therapy in Japan.

**Results:**

A small peak for the 351-keV γ ray of ^211^Bi originating from the exhalation of ^219^Rn was observed. Using the observed γ peak of ^211^Bi, the average amounts of ^219^Rn per unit breath volume at 1 min and 5 min after the start of ^223^RaCl_2_ administration were calculated as 90 ± 56 Bq/mL and 28 ± 9 Bq/mL, respectively. The effective dose of ^219^Rn to caregivers was estimated to be 3.5 μSv per injection.

**Conclusions:**

The amount of ^219^Rn in the exhaled breath of patients treated with ^223^RaCl_2_ was quantitatively calculated using breath collection bags. The internal radiation exposure of caregivers from ^219^Rn in the exhaled breath of patients treated with ^223^RaCl_2_ is relatively small.

## Background

**Fig. 1:**
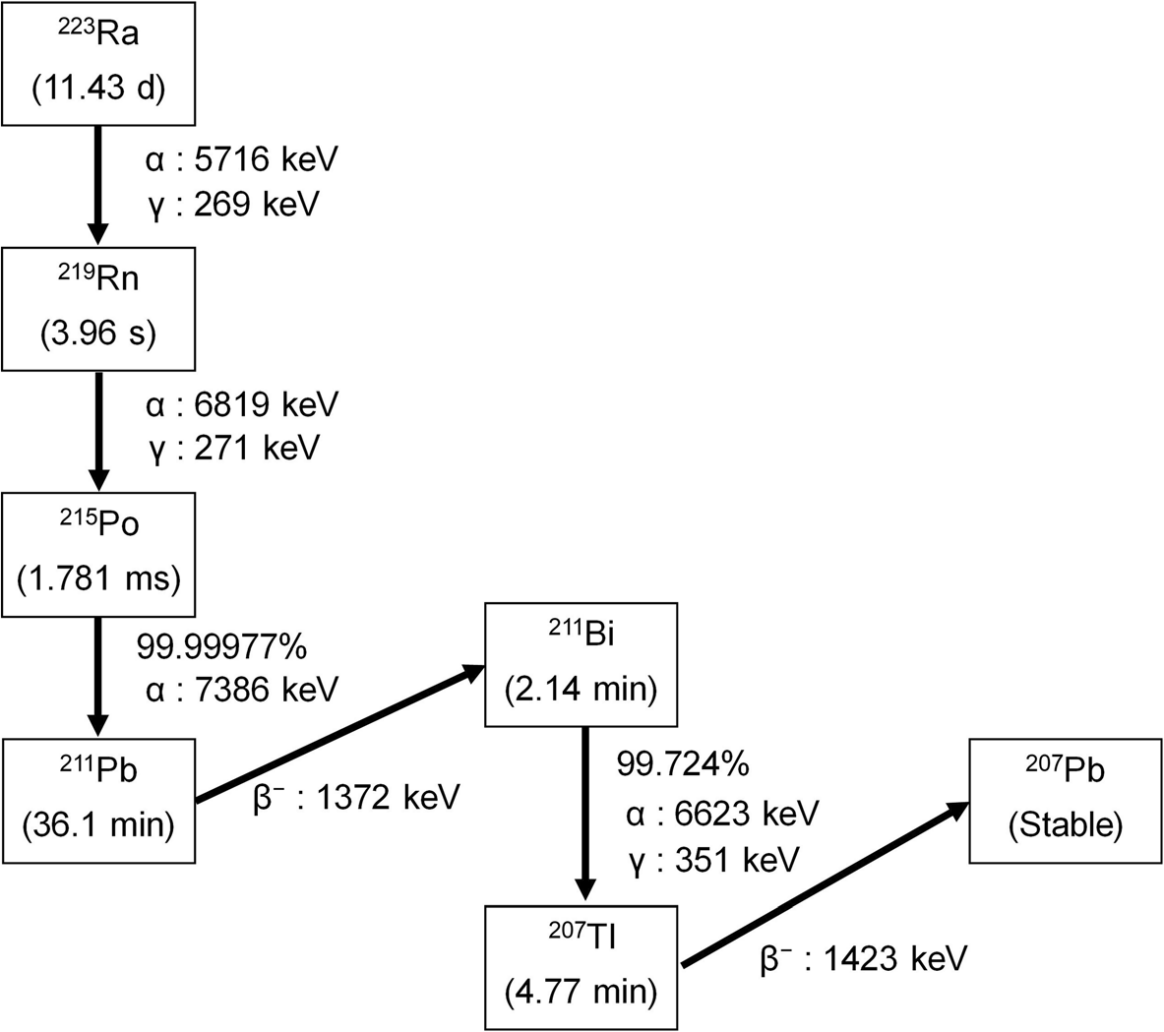
Main decay chain of ^223^Ra

Recently, radium-223 (^223^Ra) dichloride (^223^RaCl_2_; Xofigo®) has been widely used for the treatment of bone metastases in patients with castration-resistant prostate cancer (CRPC) [1, 2]. This radionuclide emits α-particles within a short range in tissues and with a high linear energy transfer, making it suitable for the treatment of cancer cells with minimal side effects in the surrounding tissues. As shown in the decay chain of ^223^Ra (Fig. 1), some descendant nuclides of ^223^Ra also emit α-particles and contribute to its killing effect on cancer cells. However, ^223^Ra decays into radon-219 (^219^Rn) which is an isotope of a noble-gas element, and this ^219^Rn may escape from patients treated with ^223^Ra. Although the escape of ^219^Rn from patients is probably minor because of the very short half-life of ^219^Rn (3.96 s), the amount of ^219^Rn escaping from patients should be quantified to allow the internal radiation exposure experienced by medical staff and caregivers to be estimated. Yamamoto et al. mentioned that ^219^Rn may escape via the respiration of patients and reported that the concentration of the descendant nuclides of ^219^Rn in a room increased during radionuclide therapy with ^223^Ra, compared with that without the patients [3]. However, the amount of ^219^Rn exhaled through the respiration of patients has not been quantified in a hospital setting. In this study, the breath of patients treated with ^223^Ra was collected using a breath collection bag and the ^219^Rn radioactivity was quantified by measuring the γ rays of ^211^Bi, which is a descendant nuclide of ^219^Rn, persisting in the breath bag. The purpose of this study was to evaluate the level of ^219^Rn radioactivity in the exhaled breath of patients treated with ^223^Ra to enable a precise estimation of the internal radiation exposure dose experienced by caregivers.

## Methods

### Measurement of expired gas from patients

The study protocol was approved by the institutional review board of Osaka University Hospital. First, we investigated whether the breath of the patients contains radionuclides emitting α-particles using an α survey meter (Hitachi TCS-232b, Tokyo, Japan). A patient (body weight 55.0 kg) received ^223^RaCl_2_ of 3.0 MBq (55 kBq/kg) for the treatment of bone metastases from CRPC. After informed consent was obtained from the patient, the α survey meter was placed closely to the mouth of the patient from the start of ^223^RaCl_2_ administration, and α-particles in the breath of the patient were directly measured until 3 min after the start of administration. Additionally, the count rate at 5 min after the start of administration was also measured.

**Fig. 2:**
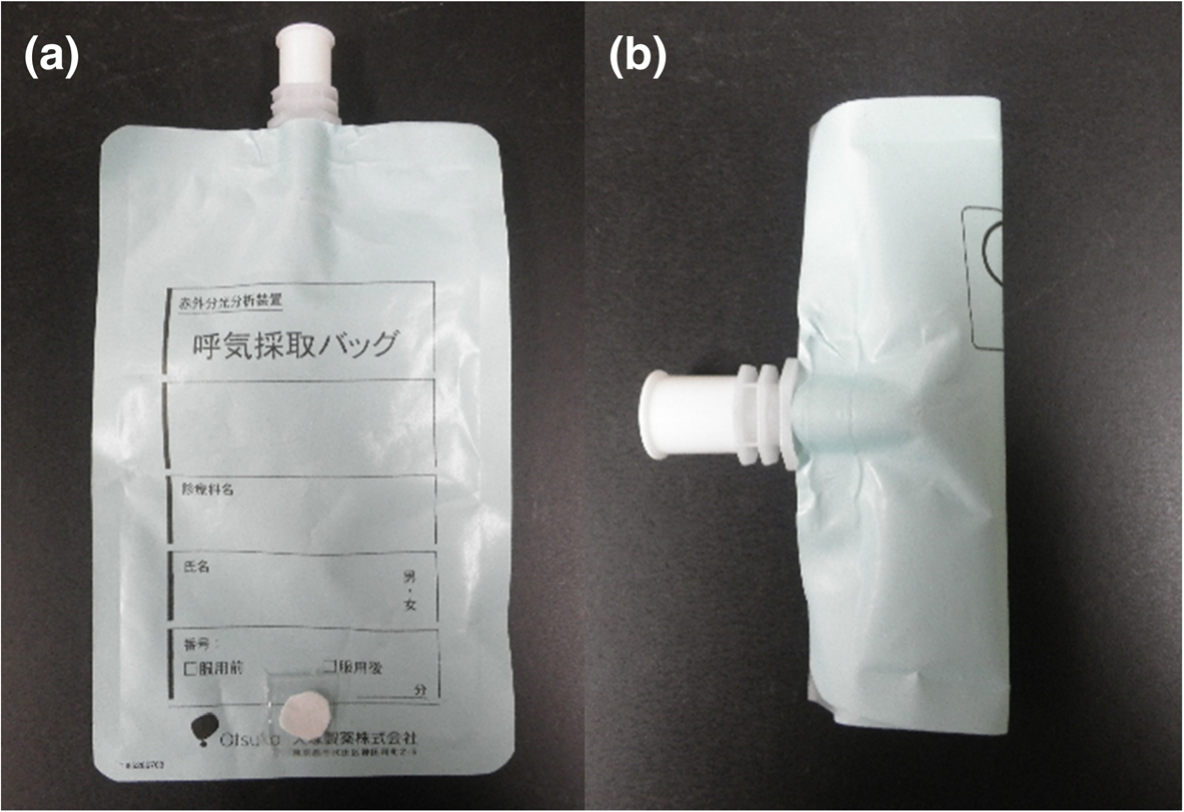
Photographs of **a** the breath bag with the glass-fiber filter after the removal of the breath sample and **b** the folded breath bag for the measurement with an HPGe detector

**Fig. 3:**
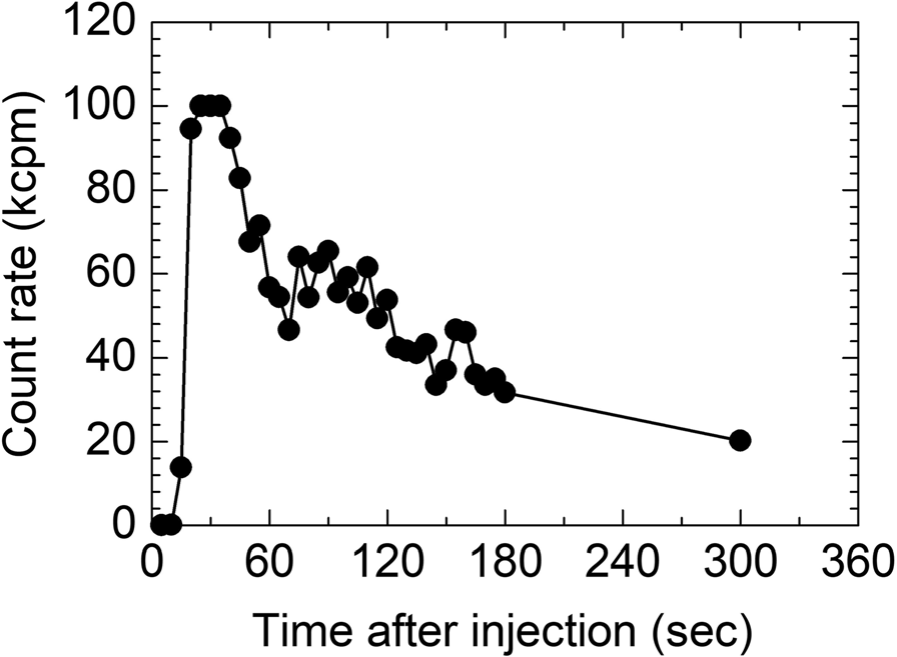
Time activity curve obtained in direct measurement of α-particles in breath of patient by an α survey meter from the start of ^223^RaCl_2_ administration. The count rate exceeded the measurable range (100 kcpm) at several tens of seconds after the start of administration

Then, a total of 12 breath samples were collected using breath collection bags (Otsuka Pharmaceutical, Tokyo, Japan) from a total of six patients (71.3 ± 5.4 years old, body weight 59.5 ± 11.7 kg) received ^223^RaCl_2_ (3.4 ± 0.8 MBq). The administration of ^223^RaCl_2_ to the patients was conducted slowly over a period of approximately 1 min. After informed consent was obtained from each patient, approximately 300 mL of exhaled breath was collected into a breath bag at 1 min and at 5 min after the start of ^223^RaCl_2_ administration. A breath sampling time of 1 min after the start of administration was selected because the amount of ^219^Rn contained in a patient’s breath is expected to reach a peak value at this time because of the slow administration of ^223^RaCl_2_ (see also Fig. 3 which is the result of the direct measurement of the breath of the patient by the α survey meter). A sampling time of 5 min is expected to correspond to the time at which the patient is likely to leave the injection room. After sampling, the breath sample was removed from the bag using a syringe (volume 50 mL, minimum volume scale 1 mL) to enable the bag to be folded into a small, fixed geometric shape (see also Fig. 2) for measurement using a high-purity germanium (HPGe) detector (Canberra BE-2020, Connecticut, USA). The volume of the breath collected in the bag was measured using the volume scale printed on the syringe (the uncertainty for the measured volume of the breath sample was estimated to be several milliliters). Because ^219^Rn and its descendant nuclides may escape from the bag during the use of the syringe, the bag was allowed to stand for more than 80 s until almost all the ^219^Rn atoms in the breath bag had decayed into non-volatile descendant nuclides prior to the removal of the breath sample. Then, the breath sample was removed through a glass-fiber filter (ADVANTEC GB-100R, Tokyo, Japan) attached to the syringe to catch the descendant nuclides of ^219^Rn. After the removal of the breath sample, the breath bag, together with the glass-fiber filter, was subjected to γ-ray spectrometry using an HPGe detector for 1800 s at almost zero distance. The amount of ^219^Rn in the breath sample was estimated as follows. Based on the observed γ peak of ^211^Bi (351 keV), the radioactivity of ^211^Pb in transient radioactive equilibrium with ^211^Bi was calculated [4]. Then, from the ^211^Pb radioactivity at the end of the breath collection, the amount of ^219^Rn contained in the breath sample was estimated assuming that the number of ^211^Pb atoms at the end of the breath collection would be equivalent to that of ^219^Rn because of the very short half-life of ^219^Rn. The measurement efficiency for the 351-keV γ peak of ^211^Bi at the same distance with the measurement of the breath bag was evaluated based on the measurement of a self-produced ^223^Ra point source, the radioactivity of which was determined using a ^133^Ba standard source. The measurement efficiency was determined to be 2.63 ± 0.13%. The uncertainty for the efficiency is including the uncertainty of the radioactivity of the ^133^Ba standard source (4.8%) and counting errors for the measurements of the ^133^Ba standard source and the ^223^Ra point source (less than 1%).

### Estimation of radiation exposure experienced by medical staff

The descendant nuclides of ^219^Rn exhaled through the breath of patients may float through the air by attaching to dust or aerosol particles. These nuclides can contribute to medical staff and caregivers being exposed to radiation internally. Therefore, we also investigated whether the inhalation of descendant nuclides of ^219^Rn can be blocked by the use of a mask. Medical staff wore an N95 mask (3M 1870+, Tokyo, Japan) during the administration of ^223^Ra to the patients. After administration, the outside and inside (face side) of the mask were measured using an α survey meter (Hitachi TCS-232b, Tokyo, Japan). A total of five masks worn by medical staff who stood close to the patients during ^223^RaCl_2_ therapy were measured during two injections (three medical staff for one injection [administration dose 3.9 MBq] and two staff for the other [administration dose: 3.0 MBq]). The measurements using the survey meter were conducted while placing the survey meter in close contact with the surface of each mask, and the count rate was recorded at 2 min after the start of measurement with a time constant of 30 s.

### Estimation of radiation exposure experienced by caregivers

The radiation exposure experienced by caregivers was calculated based on documentation regarding the calculation of release criteria established for ^131^I therapy by the Ministry of Health and Welfare in Japan [5]. The blood concentration of ^223^Ra immediately after injection (*C*_Ra_) was estimated using the following formula:

$$ {C}_{\mathrm{Ra}}=\frac{A_{\mathrm{injection}}}{V_{\mathrm{blood}}}, $$

where *A*_injection_ is the injection dose of ^223^Ra and *V*_blood_ the blood volume in the human body. In this calculation, we used *A*_injection_ = 3.4 × 10^3^ kBq which is the average injection dose in this study and *V*_blood_ = 5.0 × 10^3^ mL, and *C*_Ra_ was estimated to be 0.68 kBq/mL. The area under the curve (AUC) for the blood concentration of ^223^Ra was obtained using the clinical trial data (phase I) for ^223^RaCl_2_ published in Japan by the Pharmaceutical and Medical Devices Agency (PMDA) (https://www.pmda.go.jp). Duration (*T*), which corresponds to the AUC up to 72 h post injection (0.674 kBq·h/mL) with the assumption that the blood concentration level immediately after the injection of ^223^Ra is maintained, was calculated using the following equation:

$$ T=\frac{\mathrm{AUC}}{C_{\mathrm{Ra}}}, $$

and *T* was estimated to be 0.99 h. The concentration of ^219^Rn in the exhaled breath was assumed to be proportional to the blood concentration of ^223^Ra. The total exhaled amount of ^219^Rn was calculated by multiplying the radioactivity in the exhaled breath immediately after the injection by a factor of 0.99 h, assuming that the respiration rate was 12 breaths per minute and the volume of breath was 500 mL per single respiration. The effective dose coefficient for the inhalation of ^219^Rn was calculated as 7.9 × 10^−9^ mSv/Bq, with reference to the ^222^Rn data (6.5 × 10^−6^ mSv/Bq) [6] corrected by the effective half-life. Regarding the exposure scenario for caregivers, the volume of the room containing the patient was estimated to be 30 m^3^, air changes were regarded as once per hour, the daily respiration rate of the caregivers was estimated to be 20 m^3^, and the caregiver was assumed to always be present in the same room as the patient based on the release criteria for ^131^I in Japan [5]. To estimate the internal exposure dose experienced by caregivers, an exposure factor of 0.5 was applied [7].

## Results

The time activity curve obtained in the direct measurement of α-particles in the breath of the patient by the α survey meter is shown in Fig. 3. The count rate of α-particles exceeded the measurable range (more than 100 kcpm) at several tens of seconds after the start of administration and then gradually decreased to 20 kcpm until 5 min after the start of administration, showing that radionuclides emitting α-particle were exhaled through the respiration.

**Fig. 4:**
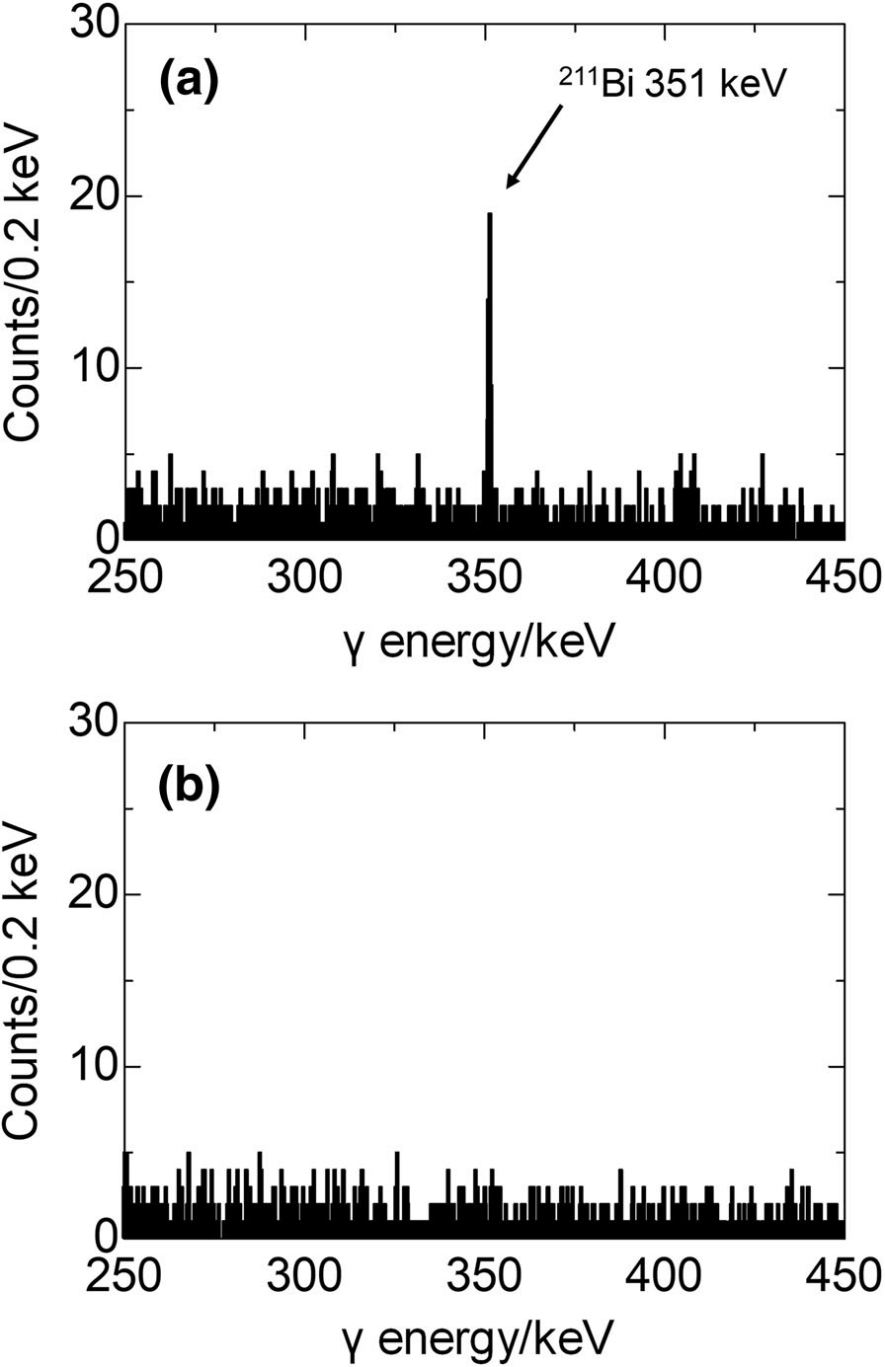
Typical γ-ray spectrum obtained for breath bag measurements using an HPGe detector. Administration dose, 3.8 MBq; breath sampling time, 5 min after the start of administration; measurement start, **a** 17 min and **b** approximately 4 h after the end of breath collection

A typical γ-ray spectra obtained for the measurement of a breath bag is shown in Fig. 4 a. A small peak for the 351-keV γ ray of ^211^Bi was observed. On the other hand, no γ peak of 269 keV from ^223^Ra was observed. The 351-keV peak disappeared approximately 4 h after the collection of the breath sample, as shown in Fig. 4 b. This finding unambiguously shows that the breath samples from patients treated with ^223^RaCl_2_ did not contain ^223^Ra.

**Fig. 5:**
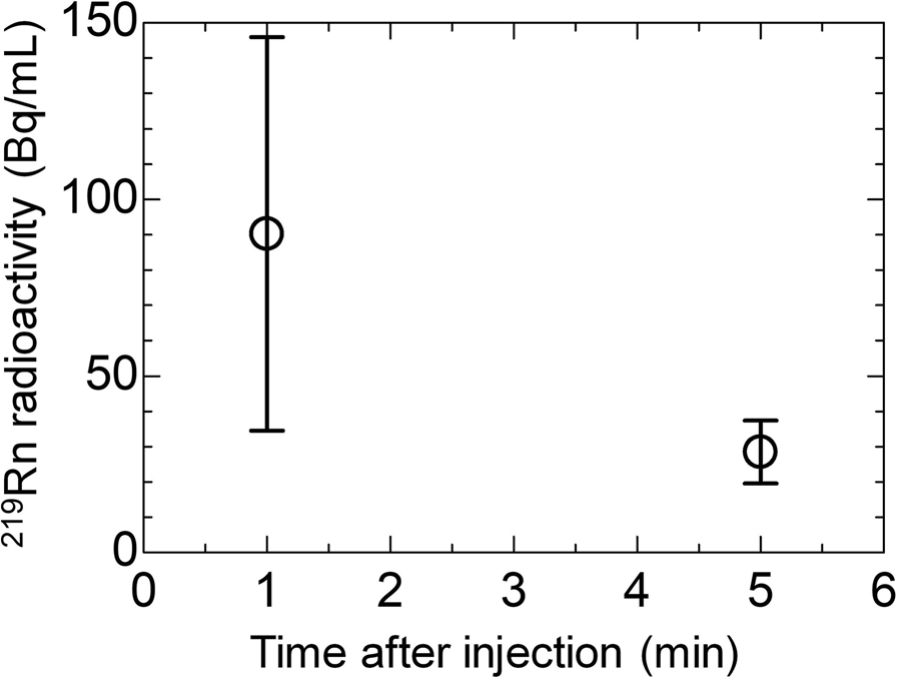
Mean value of ^219^Rn per unit volume of exhaled breath at 1 min and 5 min after the start of ^223^RaCl_2_ administration

The amount of ^219^Rn contained in the breath samples as determined using γ-ray spectrometry is summarized in Table 1, together with the administration dose of ^223^Ra and the volume of collected breath. The amount of ^219^Rn exhaled in the breath at 1 min after the start of administration varied widely from 14 to 59 kBq, while the amount of ^219^Rn at 5 min after the start of administration was around 10 kBq. The mean value of ^219^Rn per unit volume of breath is shown in Fig. 5. The values were calculated as 90 ± 56 Bq/mL and 28 ± 9 Bq/mL (mean ± standard deviation) at 1 min and 5 min after the start of administration, respectively.

**Table 1:** Summary of the amounts of ^219^Rn contained in breath samples from patients treated with ^223^RaCl_2_

Administration dose of ^223^RaCl_2_	Breath sampling time from administration start	Volume of collected breath	Activity of ^219^Rn in breath
2.5 MBq	1 min	293 mL	25 ± 4 kBq
2.5 MBq	5 min	384 mL	7.8 ± 1.4 kBq
2.5 MBq	1 min	349 mL	59 ± 6 kBq
2.5 MBq	5 min	329 mL	13 ± 2 kBq
3.2 MBq	1 min	294 mL	14 ± 3 kBq
3.2 MBq	5 min	284 mL	8.4 ± 1.5 kBq
3.8 MBq	1 min	339 mL	19 ± 3 kBq
3.8 MBq	5 min	329 mL	9.1 ± 1.4 kBq
3.9 MBq	1 min	299 mL	45 ± 5 kBq
3.9 MBq	5 min	297 mL	11 ± 2 kBq
4.3 MBq	1 min	418 mL	16 ± 3 kBq
4.3 MBq	5 min	344 mL	5.8 ± 1.3 kBq

The results of the mask measurements using the α survey meter are summarized in Table 2. Small count rates of less than 100 cpm for α-particles were detected on the outside of the masks. On the other hand, no α-particles were detected on the inside of the masks. The count rate on the outside of the masks varied widely depending on the medical staff, and the measurement for one mask showed no detection of α-particles even on the outside of the mask.

**Table 2:** Summary of measurement results obtained using an α survey meter for N95 masks worn by medical staff

Administration dose of ^223^RaCl_2_	Measured mask	Measured side	Measurement start time from administration	Observed count rate	^211^Bi radioactivity calculated based on count rate
3.9 MBq	a	Outside	40 min 7 s	8 cpm	0.8 Bq
	b	Outside	42 min 22 s	53 cpm	5.2 Bq
	b	Inside	49 min 16 s	0 cpm	0 Bq
	c	Outside	77 min 40 s	26 cpm	2.6 Bq
	c	Inside	80 min 0 s	0 cpm	0 Bq
3.0 MBq	d	Outside	7 min 0 s	0 cpm	0 Bq
	d	Inside	10 min 0 s	0 cpm	0 Bq
	f	Outside	24 min 48 s	80 cpm	7.9 Bq
	f	Inside	27 min 5 s	0 cpm	0 Bq

The cumulative amount of exhaled ^219^Rn from the patient via exhaled breath until 72 h after the start of administration (*A*_Rn,cumulative_) was calculated as follows:

$$ {A}_{\mathrm{Rn},\mathrm{cumulative}}={A}_{\mathrm{Rn},\mathrm{breath}}\times {V}_{\mathrm{breath}}\times {N}_{\mathrm{respiration}}\times T, $$

where *A*_Rn, breath_ is the amount of ^219^Rn contained in the unit volume of exhaled breath, *V*_breath_ the volume of breath per single respiration (500 mL/respiration), *N*_respiration_ the respiration rate per min (12 respiration/min) and *T* the duration with the assumption that the blood concentration level immediately after the injection of ^223^Ra is maintained (0.99 h). Using 90 ± 56 Bq/mL as *A*_Rn,breath_, which is the amount of ^219^Rn contained in the breath at 1 min after the start of administration, *A*_Rn,cumulative_ was calculated as 32 MBq. By referring to the release criteria for patients treated with ^131^I, the amount of ^219^Rn inhaled by caregivers (*A*_Rn,inhaled_) was estimated using the following formula:

$$ {A}_{\mathrm{Rn},\mathrm{inhaled}}={A}_{\mathrm{Rn},\mathrm{cumulative}}\times \frac{V_{\mathrm{daily}}}{V_{\mathrm{room}}\times {N}_{\mathrm{air}\ \mathrm{change}}}, $$

where *V*_daily_ is the daily respiration rate of the caregivers (20 m^3^/d), *V*_room_ the volume of the room containing the patient (30 m^3^) and *N*_air change_ the air changes per hour (1/h); *A*_Rn,inhaled_ was calculated as 0.89 MBq. The effective dose of ^219^Rn to caregivers (*D*_eff_) was estimated as follows:

$$ {D}_{\mathrm{eff}}={A}_{\mathrm{Rn},\mathrm{inhaled}}\times E\times F, $$

where *E* is the effective dose coefficient for the inhalation of ^219^Rn (7.9 × 10^−9^ mSv/Bq) and *F* the exposure factor (0.5 [7]); *D*_eff_ was estimated to be 3.5 μSv per injection.

### Discussion

This study showed that the amount of ^219^Rn contained in the breath of patients can be quantitatively measured using an HPGe detector based on the detection of the γ peak of the descendant nuclide ^211^Bi and a breath collection bag. In addition, the internal radiation exposure of caregivers from ^219^Rn was estimated to be as small as 3.5 μSv per injection. The high count rate of α-particles observed in the direct measurement of the breath of the patient by the α survey meter would be attributed to ^219^Rn, not to the descendant nuclides of ^219^Rn, exhaled through the respiration because the average radioactivity of ^211^Bi measured using the HPGe detector was in the order of 10 Bq and the count rate of α-particles from ^211^Bi in the measurement with α survey meter would be quite low compared with that from ^219^Rn (in the order of 10 kBq). Although the high count rate of α-particles from ^219^Rn exhaled through the respiration was observed using the α survey meter, the quantitative measurement of the descendant nuclide of ^219^Rn using the HPGe detector in this study clearly shows that the effective dose of ^219^Rn to caregivers is small. The amount of ^219^Rn contained in the breath of a patient at 1 min after the start of administration varied widely, as is shown in Table 1. The reason for this variation can be attributed to the peak shift in the exhalation of ^219^Rn from the breath of patients caused by the manual administration of ^223^RaCl_2_.

Since the effective dose coefficient for inhaled ^219^Rn has not been reported, the value for ^219^Rn was estimated based on the effective dose coefficient of ^222^Rn (6.5 × 10^−6^ mSv/Bq) [6]. α-Particles emitted from the descendant nuclides are thought to be the main contributors to the effective dose coefficient because their radiation weighting factor is very high compared with those of electrons and γ rays. Since the descendant nuclides of ^219^Rn would decay before clearance because of their short half-lives, analogous to those of ^222^Rn [8], and the total α-particle energy from the descendant nuclides of ^219^Rn would be roughly the same as that of ^222^Rn, the effective dose coefficient of ^219^Rn can be estimated by correcting the effective dose coefficient of ^222^Rn with its effective half-life. Using the biological half-life of 55 min for Rn [9] (effective half-lives of 3.95 s for ^219^Rn and 54.5 min for ^222^Rn), the effective dose coefficient of ^219^Rn was estimated to be 7.9 × 10^−9^ mSv/Bq (= 6.5 × 10^−6^ mSv/Bq × 3.95 s/54.5 min).

For the mask measurements, the observed α-particles were considered to have originated from ^211^Bi, which was in transient radioactive equilibrium with a ^211^Pb parent (half-life 36.1 min) because the half-life of ^219^Rn is very short. The N95 mask was capable of catching the descendant nuclides of ^219^Rn, that had attached to dust or aerosol particles, on the surface of the mask, but it was not thought to be capable of blocking the inhalation of ^219^Rn. If ^219^Rn had passed through the N95 mask, α-particles from the descendant nuclides of ^219^Rn that had decayed during and after passing through the mask would have been detectable on the inside of the mask. However, α-particles were not detected on the inside of the masks in the present study. Therefore, ^219^Rn is considered to have not reached the mouths of the medical staff. The reason for the variation in the count rate observed for measurements made for the outside of the masks, as shown in Table 2, is probably due to the change in the amounts of ^219^Rn and its descendant nuclides reaching the medical staff via the airflow in the injection room. The radioactivity of ^211^Bi caught by the mask, as calculated based on the measured count rate of α-particles, is expected to correspond to less than 10 Bq at the measurement start time using the instrument efficiency of 35.1%/2π written in the calibration certificate of the survey meter and an α branch of 99.724% for ^211^Bi. Although the radioactivity of the descendant nuclides of ^219^Rn caught by the mask was relatively small, wearing the N95 mask appeared to protect the internal exposure of the medical staff.

The effective dose from the inhalation of ^219^Rn experienced by caregivers was estimated to be 3.5 μSv per patient injection. This value is significantly lower than the dose of 5 mSv per episode for caregivers recommended by the International Commission on Radiological Protection (ICRP) [10, 11]; therefore, the internal radiation exposure from ^219^Rn should not affect the release criteria for patients who have been treated with ^223^RaCl_2_ in Japan.

## Conclusions

The amount of ^219^Rn contained in the breath of patients treated with ^223^Ra was determined using γ-ray spectrometry with an HPGe detector and breath collection bags. Although a small peak for γ rays of ^211^Bi originating from ^219^Rn contained in the breath was observed, the estimated internal radiation exposure to caregivers via the inhalation of ^219^Rn was small and should not affect the release criteria of patients who have been treated with ^223^RaCl_2_.

## Data Availability

The datasets generated during and/or analyzed during the current study are available from the corresponding author on reasonable request.

## Abbreviations

AUC: Area under the curve
CRPC: Castration-resistant prostate cancer
HP: High purity
ICRP: International Commission on Radiological Protection
PMDA: Pharmaceutical and Medical Devices Agency
